# Facial Expression Influences Face Identity Recognition During the Attentional Blink

**DOI:** 10.1037/a0037945

**Published:** 2014-10-06

**Authors:** Dominik R. Bach, Martin Schmidt-Daffy, Raymond J. Dolan

**Affiliations:** 1 Wellcome Trust Centre for Neuroimaging, University College London, and Department of Psychiatry, Psychotherapy, and Psychosomatics, University of Zurich; 2 Department of Psychology and Ergonomics, Berlin Institute of Technology; 3 Wellcome Trust Centre for Neuroimaging, University College London

**Keywords:** facial expression of emotion, visual search, automatic processing, attentional capture, threat detection

## Abstract

Emotional stimuli (e.g., negative facial expressions) enjoy prioritized memory access when task relevant, consistent with their ability to capture attention. Whether emotional expression also impacts on memory access when task-irrelevant is important for arbitrating between feature-based and object-based attentional capture. Here, the authors address this question in 3 experiments using an attentional blink task with face photographs as first and second target (T1, T2). They demonstrate reduced neutral T2 identity recognition after angry or happy T1 expression, compared to neutral T1, and this supports attentional capture by a task-irrelevant feature. Crucially, after neutral T1, T2 identity recognition was enhanced and not suppressed when T2 was angry—suggesting that attentional capture by this task-irrelevant feature may be object-based and not feature-based. As an unexpected finding, both angry and happy facial expressions suppress memory access for competing objects, but only angry facial expression enjoyed privileged memory access. This could imply that these 2 processes are relatively independent from one another.

Emotional facial expression is an important social signal, and there is considerable evidence for prioritized processing, particularly when it is negatively valenced (Bar-Haim, Lamy, Pergamin, Bakermans-Kranenburg, & van IJzendoorn, 2007). This has been shown as increased detection speed for angry faces in a face-in-the-crowd task (Horstmann & Bauland, 2006; Schmidt-Daffy, 2011), preferential spatial attention for arousing facial expression in a dot probe task (Macleod & Mathews, 1988; MacLeod, Mathews, & Tata, 1986), and privileged memory access for angry and fearful expression when capacity is limited in the attentional blink task (de Jong, Koster, van Wees, & Martens, 2009; Fox, Russo, & Georgiou, 2005; Luo, Feng, He, Wang, & Luo, 2010; Maratos, Mogg, & Bradley, 2008; Milders, Sahraie, Logan, & Donnellon, 2006). The latter comprises a rapid serial visual presentation (RSVP) stream with two embedded targets (T1, T2)—for example, letters, words, or pictures. If T1 and T2 are separated by a few distractor items, presence or identity of the T2 is less well reported than if it follows the T1 either immediately, or after an interval longer than 500–800 ms, a phenomenon termed *attentional blink* (AB) (Chun & Potter, 1995; Isaak, Shapiro, & Martin, 1999; Raymond, Shapiro, & Arnell, 1992). During this AB period, arousing T2 words are less susceptible to the blink phenomenon than nonarousing ones (Anderson, 2005; Anderson & Phelps, 2001; De Martino, Kalisch, Rees, & Dolan, 2009; Keil & Ihssen, 2004), in line with an account that the “blink” phenomenon occurs between target detection and memory encoding (Dux, Ivanoff, Asplund, & Marois, 2006). It thus reflects impaired access to memory, but not impaired initial (possibly preconscious) detection. Similarly, the presence of angry and fearful T2 faces is better recalled, and their expression better remembered, than neutral T2 (de Jong et al., 2009; Fox et al., 2005; Luo et al., 2010; Maratos et al., 2008; Milders et al., 2006). Evidence is somewhat less clear for happy expression (de Jong et al., 2009; Fox et al., 2005; Mack, Pappas, Silverman, & Gay, 2002; Miyazawa & Iwasaki, 2010), and there is a suggestion of privileged memory access for angry as compared to happy facial expression in T2 position (de Jong & Martens, 2007). In addition, angry faces in T1 position suppress T2 recognition (de Jong, Koster, van Wees, & Martens, 2010; Maratos, 2011), suggesting prioritized processing of an angry face in T1 position as well.

These observations indicate that negative, and possibly also positive, facial expressions capture attention. It is striking that all aforementioned studies have tasked participants to remember the emotional expression of the targets. An important question is whether and how emotional facial expression impacts memory access when it is task-irrelevant and instead face identity is relevant for the task. It has been proposed that features that are close together in time or space form objects, and that attention is, as a rule, directed toward objects and not toward individual features (Mather, 2007). This possibility has been addressed for emotional words and nonfacial pictures where task-irrelevant arousing distractors presented close to a neutral target stimulus in a RSVP suppress memory for the target, a phenomenon termed *emotional AB* (McHugo, Olatunji, & Zald, 2013; Most, Chun, Widders, & Zald, 2005). Other experiments have demonstrated that when colored words with task-irrelevant semantic meaning are embedded in a RSPV and the task is to report the color, performance is better for emotional compared to neutral words (Arend, Botella, & Barrada, 2003). What both types of experiments demonstrate is attentional capture by task-irrelevant features. Importantly, in one case, an arousing, task-irrelevant feature grabs attention and a task-relevant feature is suppressed while in the other case a concomitant task-relevant feature is prioritized. This discrepancy is resolved by recognizing that in the first case, the task-relevant feature belongs to a different object, and in the second case, it belongs to the same object. A framework of object-based attention predicts that if a salient feature captures attention, then the entire object enjoys privileged memory access, whereas features of other objects are likely to be suppressed (Mather, 2007). This framework predicts that attentional capture by emotional facial expression would also improve identity processing of the same face.

In the present study, we addressed attentional capture by task-irrelevant features for angry and happy facial expression. First, we were interested whether emotional expression in angry or happy face photographs can capture attention at all when it is task-irrelevant. In fact, for fearful faces there is mixed evidence (Milders et al., 2006; Stein, Zwickel, Ritter, Kitzmantel, & Schneider, 2009), and it is suggested that when they are task-relevant, attentional capture is not automatic but can be reduced by increasing distraction (Stein, Peelen, Funk, & Seidl, 2010). In a visual search task, attentional capture by irrelevant facial expression has been established for happy, but not angry or fearful expression (Hodsoll, Viding, & Lavie, 2011). Hence, it remains an open question whether irrelevant angry or happy facial expression in T1 and T2 position can capture attention in the AB setup. Second, we were interested in whether the impact of attentional capture is based on objects or on features. In the attentional blink paradigm, T1 and T2 are two clearly separable objects; in our implementation they are face photographs with different identity. This predicts that if attentional capture is based on objects, emotional expression of one target should enhance reporting the identity of the same target, and suppress reporting identity of the other target. However, if attentional capture is based on features, then emotional expression of one target should suppress reporting identity of the same and of the other target.

## Methods

### Subjects

Forty female university students (*M* age ± *SD* = 26.9 ± 3.5 years) volunteered for Experiment 1, 20 students (eight male, 12 female, 24.6 ± 5.0 years) for Experiment 2, and 24 students (seven male, 17 female, 23.2 ± 3.8 years) for Experiment 3; all samples were independent from one another. The study was approved by the local research ethics committee.

### Design and Independent Variables

Experiment 1 followed a 3 (T1 valence) × 3 (T2 valence) × 5 (T1-T2 lag) factorial design, and Experiments 2 and 3 followed a 3 (T1 [Exp. 3] or T2 [Exp. 2] valence) × 10 (T1-T2 lag) factorial design. In one experimental block, three male faces appeared as T1 and three female faces as T2, and vice versa in the second block, whereas the sequence of the two blocks was balanced across participants. For each cell of the design (trial type), each of three actors for T1 and T2, respectively, was presented twice per block. In Experiment 1 we were mainly interested in trials with neutral T1 (15 trial types, 180 trials). Therefore, each actor was only presented once per block in other trial types (30 trial types, 180 trials). This procedure resulted in overall 360 trials for each of the three experiments. Trials were intermixed randomly, and T1-T2 actor combinations in single trials were varied randomly across subjects. In Experiments 2 and 3, we added an additional 36 single-target trials, presenting each of the six actors with three facial expressions twice. We report trials with the same expression as the T2 targets for comparison with the two-target trials.

### Dependent Variables

After each trial, participants were tasked to recognize the identity of the faces previously shown as T1 and T2, respectively, by choosing one out of three simultaneously presented faces (all with the same sex and emotional expression as T1 and T2, respectively). Dependent variable for all analyses was T2 recognition, averaged across all trials where T1 was recognized correctly.

### Apparatus

Experiment 1 was programmed in e-prime (Psychology Software Tools, Sharpsburg, PA) and presented on a 14-in LCD screen. Experiments 2 and 3 were programmed in Cogent 2000 (www.vislab.ucl.ac.uk) and presented on a 20-in LCD screen, using a chin rest for constant viewing distance. Screen refresh rate was 60 Hz and screen resolution 1024 × 768 pixels for all experiments.

### Stimuli

**Figure 1 fig1:**
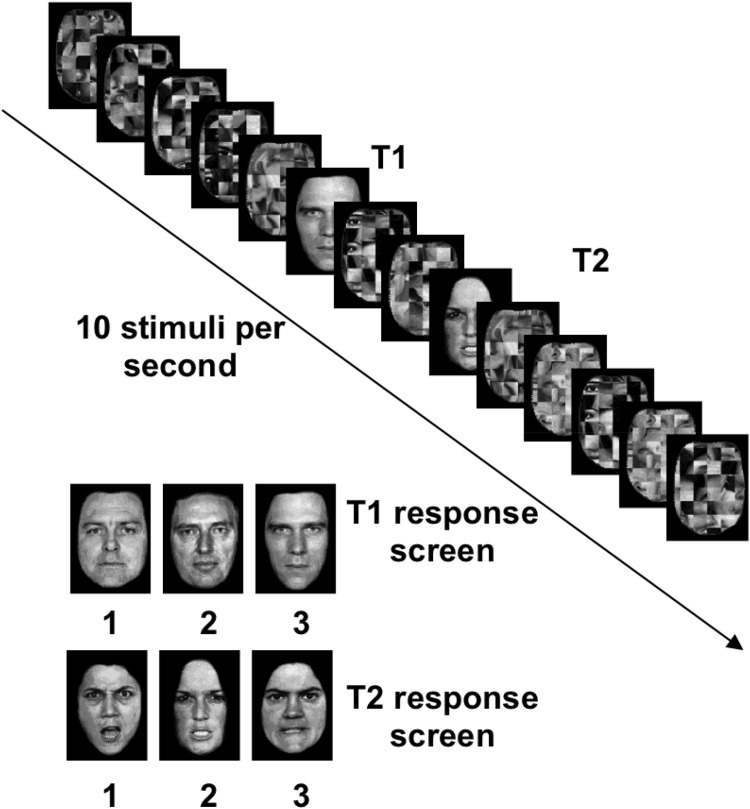
Intratrial sequence: Two face targets (T1, T2) are embedded in a 10 Hz rapid visual serial presentation of scrambled faces. In this example, T2 occurs after two distractors, this is, at Lag 3. After each trial, three possible face identities are shown for T1 and T2, respectively.

Face stimuli (see example in Figure 1) were modified pictures of facial affect (Ekman & Friesen, 1976). Details about stimulus construction are given in (Schmidt-Daffy, 2011). Distractor stimuli were constructed by cutting pictures of facial affect into squares of 5 × 10 pixels, randomly combining them and applying the background mask of target stimuli (see Figure 1). All stimuli were presented on black background with a size of 74 × 101 pixel (visual angle of 1.72 ×2.29).[Fig-anchor fig1]

### Procedure

Each experiment consisted of 25 practice trials, followed by two blocks of 180 trials each. After a fixation cross (1,000 ms), face stimuli were shown at 10 Hz (4 frames/∼67 ms stimulus, 2 frames/∼33 ms blank). Five, 10, or 15 distractors were presented before T1. T1 and T2 were separated by 0, 1, 2, 3, or 7 distractors (Experiment 1) or 0–9 distractors (Experiments 2 and 3). After T2, five more distractors were shown. For single-target trials in Experiments 2 and 3, a single target was shown after five, 10, or 15 distractors and was followed by five distractors. After the trial, the three possible T1 identities and the three possible T2 identities were presented on two subsequent screens, from which participants selected with a key press.

### Data Analysis

For each subject, T2 response accuracy was extracted and averaged across stimuli for all trials on which T1 was correctly identified. Statistical analyses were performed with SPSS, using a 3 × 5 (Experiment 1), 3 × 3 × 5 (Full model, Experiment 1) or 3 × 10 analysis of variance. Results are stated in Table 1, and post hoc tests as well are stated in the main text. In an exploratory approach, T1 recognition was tested in a univariate 3 × 10 analysis of variance. Results are stated in the main text.[Table-anchor tbl1]

## Results

### Experiment 1

#### Recognition of valence-varied T2 after neutral T1

**Figure 2 fig2:**
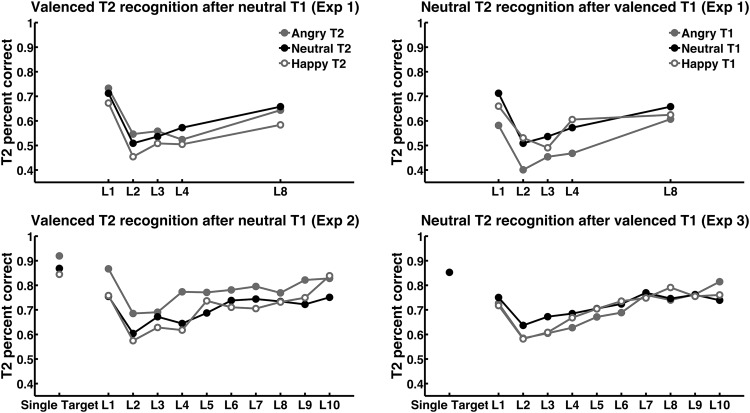
Target 2 (T2) recognition percentage after correctly recognized Target 1 (T1), for the three experiments. Analysis of variance results are summarized in Table 1. L: Lag.

T1-T2 lag impacted the recognition of valence-varied T2 after correctly recognized neutral T1, as standardly observed in AB tasks, with a U-shaped time course (Table 1, Figure 2). As hypothesized, T2 valence had a significant impact on T2 recognition. There was no interaction with lag. Post hoc contrasts revealed that happy face identity was recognized less well than neutral, *F*(1, 39) = 11.3, *p* < .01, or angry, *F*(1, 39) = 9.1, *p* < .01, face identity. T2 recognition at Lag 8 did not recover to the level of Lag 1 across all T2 (contrast Lag 8 vs. Lag 1: all T2, *F*(1, 9) = 7.3, *p* < .01), implying an AB period that is longer than reported previously.[Fig-anchor fig2]

#### Recognition of neutral T2 after valence-varied T1

T1-T2 lag had a highly significant impact on the recognition of neutral T2 after correctly recognized valence-varied T1 (see Table 1). There was no difference between Lag 1 and Lag 8 recognition across all T1 or for any of the T1 valence categories. Post hoc contrasts revealed that angry T1 expression impaired T2 recognition compared to neutral, *F*(1, 39) = 13.0, *p* < .001, and happy, *F*(1, 39) = 8.1, *p* < .01, expression. There was no interaction with lag.

#### Full model

A full model, including combinations of valence-varied T1 and valence-varied T2 confirmed the impact of lag (see Table 1). Across all combinations, there was no performance difference between Lags 1 and 8. We observed a significant impact of T1 but not of T2 valence on T2 recognition and a trend-level interaction of T1 valence and lag. T2 recognition was impaired after angry T1 for all lags, and after happy T1 for medium lags alone.

Taken together, this experiment confirms the known time course of the AB for the identification of valence-varied faces, with the exception that recognition at Lag 8 did not fully recover after a neutral T1. We observed an impact both of T1 and T2 valence on T2 recognition when analyzing combinations in which at least one target was neutral. In a full model, the effect of T1 was clearly dominant. We did not observe a significant interaction of target valence and lag, indicating that the valence effects might not arise from capacity limits during the AB period. However, because finding an interaction relies on using lags after the AB period, and because the AB period in this experiment appeared to be longer than Lag 8 for some target combinations, this observation might also be due to a lack of power. Hence, Experiments 2 and 3 were conducted to more fully sample lags from within and outside the AB period and to investigate valence-varied target recognition in the absence of interfering items, that is, in single-target trials. Also, we separated manipulation of T1 and T2 valence into two separate experiments to increase the number of trials per condition, reduce noise in the averages, and thus enhance statistical power.

### Experiment 2

This experiment was designed to separately assess the impact of T2 facial expression on T2 identity recognition after neutral T1 without interference from other trial types, using 10 lags, and 12 trials per cell of the design, thereby doubling the number of trials per cell, and hence, sensitivity. We hypothesized a replication of the T2 valence effect observed in Experiment 1.

#### Valence-varied T2 recognition after neutral T1

Lag impacted recognition of valence-varied T2 after neutral T1 (see Table 1). Recognition at Lags 1 and 10 was not different for any T2 valence, or across all T2 valences, indicating that we fully sampled the entire AB period. T2 valence significantly influenced T2 recognition, which was better for angry than for happy, *F*(1, 19) = 20.8, *p* < .001, or neutral T2, *F*(1, 19) = 9.5, *p* < .01. There was no interaction of T2 valence and lag. A similar, trend-level significant impact of target valence was found on single target recognition, *F*(2, 38) = 3.0, *p* = .077. Hence, we corrected T2 recognition in AB trials for the single-trial recognition, after which the T2 valence effect vanished, *F*(2, 38) < 1, n.s.). Therefore, the impact of T2 valence in this experiment was not dependent on the specific capacity limits during the AB.

#### T1 recognition

The AB paradigm is designed to measure impairment in T2 recognition with generally much higher global recognition rates for T1 than for T2. Nevertheless, we analyzed T1 recognition in an exploratory approach. Both for global T1 recognition and for T1 recognition before correctly recognized T2, T1 recognition depended on lag, global: *F*(9, 171) = 14.5, *p* < .001; before correct T2:, *F*(9, 171) = 11.7, *p* < .001, but not on T2 expression, global: *F*(2, 38) < 1, n.s.; before correct T2: *F*(2, 38) < 1, n.s.

### Experiment 3

This experiment was designed to separately assess the impact of T1 facial expression on neutral T2 identity recognition without interference from other trial types, using 10 lags, and 12 trials per cell of the design. We hypothesized a replication of the T1 valence effect on T2 identity recognition observed in Experiment 1.

#### Neutral T2 recognition after valence-varied T1

Lag impacted neutral T2 recognition after valence-varied T1 (see Table 1). Performance at Lag 10 was slightly better than at Lag 1, *F*(1, 23) = 3.9, *p* = .06. There was no main effect of T1 valence on T2 recognition, but a significant interaction of T1 valence and lag: According to post hoc contrasts, T2 recognition was worse after angry/happy T1 than after neutral T1, for Lags 2–6 compared to all other lags, *F*(1, 23) = 34.1, *p* < .001. There was no evidence for a differential effect of angry and happy T1 on T2 recognition.

#### T1 recognition

T1 recognition was analyzed in an exploratory approach. Both for global analysis of T1 and for T1 before correctly recognized T2, lag had a significant effect on T1 recognition—global: *F*(9, 207) = 30.9, *p* < .001; before correct T2:, *F*(9, 207) = 43.9, *p* < .001. Also, valence had a significant effect—global: *F*(2, 46) = 49.4, *p* < .001; before correct T2: *F*(2, 46) = 20.6, *p* < .001. Post hoc contrasts revealed that angry T1 were recognized better than neutral—global: *F*(1, 23) = 49.4, *p* < .001; before correct T2: *F*(1, 23) = 38.9, *p* < .001—or happy T1—global: *F*(1, 23) = 49.4, *p* < .001; before correct T2: *F*(1, 23) = 27.6, *p* < .001.

## Discussion

We investigated whether attentional capture by emotional facial expression occurs when task-irrelevant and whether it impacts processing of facial identity. Three main findings emerge. First, task-irrelevant emotional facial expression in T1 or T2 position influenced T2 identity recognition, suggesting it automatically captures attention. Emotional expression increased memory for face identity of the same target, but suppressed memory for face identity of other targets. This confirms predictions of an object-based framework of attentional capture by facial identity. Lastly, angry but not happy target faces enjoyed privileged identity recognition, independent from capacity limits, whereas in Experiment 3, both angry and happy expression suppressed identity recognition of the other target, dependent on capacity limits.

Our experiments confirm that attention to facial expression, and to face identity, are not separable. This is in keeping with the predictions of an object-based attention framework according to which objects are formed from basic features, and attention is directed toward objects, not toward individual features. On the other hand, a possibility that facial identity and face expression are composed of overlapping sets of basic physical features could, in theory, also explain the inseparability of facial expression and face identity. This possibility cannot be ruled out in the present study, although we note that there is no entirely convincing evidence for this in the literature.

The pattern of angry T2 advantage contrasts with the pattern of T2 suppression by arousing (angry and happy) T1 with specific AB influence in Experiment 3. Importantly, exploratory analysis of Experiment 3 demonstrated that although both angry and happy T1 equally suppressed T2 recognition, T1 recognition was only enhanced for angry but not for happy T1. The latter is the same pattern as angry T2 advantage in Experiments 1 and 2. In other words, happy faces in T1 position suppress T2 memory but do not enjoy privileged memory access themselves. This suggests that object-based attentional capture is not a unitary process in which advantage for the attention-grabbing object, and suppression of other objects, are two sides of the same coin. In fact, privileged memory access might be a process independent from suppressed memory access for other objects.

As a tentative model, we speculate that privileged memory access for angry faces depends on simple perceptual features that reliably differentiate possible threat from nonthreat. This would explain why it is relatively independent from capacity limits. In this model, privileged memory access is a direct consequence of stimulus attributes and not a consequence of processing limits. It is interesting that the anger detection advantage in the face-in-the-crowd task has already been explained with a sensory-bias hypothesis that states that particular perceptual features account for the impact of angry faces (Horstmann & Bauland, 2006). On the other hand, the arousing quality of both angry and happy faces might bind resources for processing these objects and therefore suppresses processing access for other objects at a stage before any of these objects would enter memory, but only under limits in processing resources. Reduced processing of competing objects could explain reduced access to memory, yet without necessarily causing privileged access to memory for the arousing object. Reduced memory access for competing objects, in this model, is a consequence of processing limits at stages before objects are encoded into memory. An alternative, conceptually similar explanation is that arousing facial expression does not actually suppress memory encoding of competing faces but rather interferes with retrieval of competing faces. This is because T1 is always recalled before T2—hence an arousing T1 facial expression could lead to increased processing upon retrieval and interfere with subsequent retrieval of a neutral T2. This might be supported by the observation that arousing T1 interferes with neutral T2 recognition, but arousing T2 does not interfere with neutral T1 recognition. Changing the order of retrieval might arbitrate between these two explanations.

This model critically relies on the effects of happy face stimuli in T1 position of our paradigm for which we observed an unexpected discrepancy between lack of privileged memory access and suppression of memory for other objects. Notably, although the impact of valence-varied T1 showed the same ordering in Experiments 1 and 3, the pattern described here was only observed in the better-powered Experiment 3, and this may imply a variability in this phenomenon. To corroborate the independence of privileged memory access, and suppressed memory access for other objects, it would be necessary to replicate these findings using other stimuli. Whether perceptual features of angry faces truly account for the observed privileged memory access is difficult to assess in the present paradigm. Previous work has used inverted or schematic faces to control for perceptual features, but this not only changes the emotional meaning of a face but also the perception of its identity. We hope in future work to address the question of perceptual features in a more elaborate fashion.

Previous studies have used the attentional blink paradigm to investigate attentional capture by fearful expression and have found that faces with task-irrelevant fearful expression enjoy privileged memory access in T2 position (Milders et al., 2006) but that fearful expression in T1 position suppresses T2 memory only when it is task-relevant (Stein et al., 2009). The latter findings clearly contradict automatic attentional capture. In this experiment, gender was the task-relevant dimension. Using angry, happy, and fearful face expressions in the same experiment, varying the same task-relevant dimension, and controlling for arousal of the face expression might clarify the discrepancy between this experiment and ours.

In summary, we observed attentional capture by task-irrelevant emotional expression that is broadly in keeping with an object-based attentional capture. As an unexpected finding, our data might suggest, if replicated, that privileged memory access for the attention-grabbing object, and memory suppression for competing objects, could be two independent processes.

## Figures and Tables

**Table 1 tbl1:** Lag × Valence Repeated-Measures Analysis of Variance for the Three Experiments

Effect	*df*	ε	η^2^	*F*	*p*
Experiment 1: Valence-varied T2 after neutral T1					
Lag	4,156	.995	.332	19.4	<.001
T2 valence	2,78	.998	.138	6.3	<.01
Lag × T2 valence	8,312	1.000	.010	<1	n.s.
Experiment 1: Neutral T2 after valence-varied T1					
Lag	4,148	.967	.278	14.3	<.001
T1 valence	2,74	.868	.181	8.2	<.001
Lag × T1 valence	8,296	.744	.010	<1	n.s.
Experiment 1: Full model					
Lag	4,124	1.000	.505	31.6	<.001
T1 valence	2,62	.908	.219	8.7	<.001
T2 valence	2,62	.941	.044	1.4	n.s.
Lag × T1 valence	8,248	.870	.060	2.0	.078
Lag × T2 valence	8,248	.972	.015	<1	n.s.
T1 valence × T2 valence	4,124	.902	.025	<1	n.s.
Lag × T1 valence × T2 valence	16,496	.761	.017	<1	n.s.
Experiment 2: Valence-varied T2 after neutral T1					
Lag	9,171	.877	.288	7.7	<.001
T2 valence	2,38	.704	.278	7.3	<.01
Lag × T2 valence	18,342	.690	.041	<1	n.s.
Experiment 3: Neutral T2 after valence-varied T1					
Lag	9,207	.969	.419	16.6	<.001
T1 valence	2,46	.595	.092	2.3	.10
Lag × T1 valence	18,414	.995	.069	1.7	<.05
*Note*. *p* values were computed after correcting degrees of freedom according to Greenhouse-Geisser. T1 = Target; T2 = Target 2.
